# Severe Symptomatic Hyponatremia Secondary to Escitalopram-Induced SIADH: A Case Report with Literature Review

**DOI:** 10.1155/2018/3697120

**Published:** 2018-09-05

**Authors:** Rishi Raj, Aasems Jacob, Ajay Venkatanarayan, Mohankumar Doraiswamy, Manjula Ashok

**Affiliations:** ^1^University of Kentucky, Lexington, KY 40536, USA; ^2^Monmouth Medical Center, Long Branch, NJ 07740, USA

## Abstract

Hyponatremia is a well-known medication related side effect of selective serotonin reuptake inhibitors; despite its association with escitalopram, the newest SSRI is very rare. We did a review of literature and came across only 14 reported case of this rare association of SIADH with escitalopram. We hereby report a case of a 93-year-old female who presented with generalized tonic-clonic seizure and was diagnosed with severe hyponatremia due to escitalopram-induced syndrome of inappropriate antidiuretic hormone secretion (SIADH). With this article, we want to emphasize clinicians about this rare side effect of escitalopram use and look for the risk factors leading to SIADH.

## 1. Background

The syndrome of inappropriate antidiuretic hormone secretion (SIADH) is a well-known cause of hyponatremia. It is a diagnosis of exclusion and can be secondary to pulmonary disorders, infections, malignant diseases, central nervous system disorders, or drugs [1]. Selective serotonin reuptake inhibitors (SSRIs) are a well-known cause of SIADH with a threefold increased risk compared to other antidepressants [2]. Hyponatremia from escitalopram, one of the newest SSRI is rare with only few reported cases in literature. Here, we report a case of severe hyponatremia which was associated with escitalopram. The importance of the report lies in the fact that SSRIs are one of the most widely used antidepressants and it is imperative for the clinicians to be more widely aware of the life-threatening nature of the side effect of this seemingly benign medication.

## 2. Case Presentation

A 93-year-old Caucasian female was brought to emergency room for gradual decline in mental status over a course of one week. Her past medical history was significant for coronary artery disease, hypertension, diabetes mellitus, hyperlipidemia, and mild cognitive dysfunction. Her medications included aspirin 81 mg daily, metoprolol 25 mg daily, amlodipine 10 mg daily, and atorvastatin 20 mg daily. Four days prior to the onset of symptoms, she was started on escitalopram 10 mg daily by her primary care physician for newly diagnosed depression. She had a routine blood work immediately prior to the outpatient visit and on retrospective review was found to have a serum sodium level of 136 mEq/L. Patient developed drowsiness and inability to maintain conversation after four days of starting escitalopram. Her mental status continued to gradually decline over the course of next few days and on the day of presentation, which was seven days from the onset of symptoms, she was noted to have intermittent severe agitation. She did not have fever, chills, neck rigidity, myalgia, recent trauma, or fall. There was no reported seizure activity or loss of consciousness. At the time of admission, patient was afebrile, blood pressure was 134/57 mm Hg, pulse rate was 75 beats/min, and respiratory rate of 18 and saturating was 99% on room air. Her BMI was 27.1. She was noted to be lethargic and was only responsive to painful stimuli. Mucous membranes were moist, and the skin turgor was intact. Her GCS score was 7 (E2V1M4). Neurological examination was negative for any gross focal neurological deficits. The rest of the physical examination was unremarkable. While, in the emergency room, patient was noted to have generalized tonic-clonic seizure which was controlled with a single dose of intravenous lorazepam 1 mg. Initial laboratory investigation results are presented in Table 1, along with reference range values. Notably, patient's serum osmolality was 234mosm/kg (normal range: 275–295 mosm/kg), urine osmolality was 468 mosm/kg, serum sodium was 105 mEq/L (normal range: 135–145 mEq/L), and urinary sodium was 68 mEq/L (normal range 25-150 mEq/L). Her thyroid-stimulating hormone (TSH) and early morning free cortisol were 0.8*μ*IU/mL (normal range 0.5-5.0 *μ*IU/mL) and 49.2 *μ*g/dL (normal range 1-75 *μ*g/dL), respectively. Chest X-ray as well as CT Head without contrast were unremarkable.

## 3. Diagnosis

Based on her clinical findings of witnessed seizure, laboratory findings of severe hyponatremia, hypoosmolality, elevated urine osmolality, and elevated urinary sodium in the presence of normal adrenal and thyroid function, a diagnosis of acute severe symptomatic hypotonic hyponatremia or SIADH was made based on the diagnostic criteria (Table 2). The current symptoms were attributed to initiation of escitalopram due to the temporal relation.

## 4. Treatment

**Figure 1:**
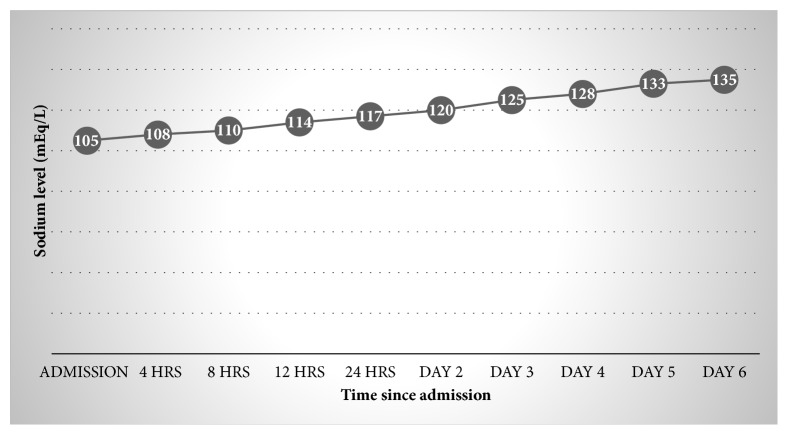
Sodium levels of the patient during the course of treatment.

Escitalopram was discontinued on the day of admission. She was started on 3% hypertonic saline at 20 ml/hr with frequent monitoring of her sodium levels with a goal sodium correction of less than 10 mEq/L in 24 hrs. Patient was put on water restriction to less than one liter per day. She was also given 2 doses of Tolvaptan 15 mg on Day 3 and Day 5 of hospitalization. Patient did not have any further episodes of seizures. Her sodium levels started to trend up and her sodium levels became normal on the 6^th^ day of hospitalization (Figure 1). The patient was finally discharged home on Day 6. Sodium levels on discharge were 135 mEq/L. She was discharged without any SSRI. Repeat sodium levels on 1 week and 2 weeks were 138 mEq/L and 142 mEq/L, respectively. She was eventually started on Mirtazapine 15 mg at bedtime on outpatient follow-up and her sodium levels remained within normal range at 3 and 6 months.

## 5. Discussion & Review of Literature

SIADH is defined as euvolemic hypotonic hyponatremia (serum sodium level of less than 135mmol/L), inappropriately elevated urine osmolality (usually more than 200 mmol/kg) relative to plasma osmolality, and an elevated urine sodium level (typically greater than 20 mmol/L) with normal renal, adrenal, and thyroid functions. Since Schwartz et al. first described it about 6 decades ago in 1957 in two patients, SIADH now has a long list of potential causes including malignant neoplasms, nonmalignant pulmonary diseases, central nervous system disorders, and drugs. In a single center retrospective study, Shepshelovich et al. compared different drug classes and showed a higher incidence of SIADH with antidepressants mainly SSRIs compared to other drug classes [3]. SSRIs are becoming increasingly leading because of SIADH with a reported incidence of 1 out of 200 patients per year [4]. SSRIs are first-line drugs for the treatment of geriatric depression owing to their safety, easy dose titration, and low rates of anticholinergic and cardiovascular adverse events [5]. Among SSRIs, escitalopram is the newest SSRI and was FDA approved in 2002. It is composed of only (S)-enantiomer of citalopram and inhibits the binding of serotonin (5-HT) to serotonin transporter (SERT), resulting in increased 5-HT concentration in synaptic cleft, which leads to increased binding of 5-HT to postsynaptic receptors causing improvement in the depression symptoms [6]. Experimental studies have revealed that enhanced serotonergic tone result in stimulation of antidiuretic hormone (ADH) secretion resulting in hyponatremia, provided water intake is sufficient [7]. As a matter of fact, since the publication of the above literature, there has been increasingly higher number of reports on escitalopram-induced SIADH. We aimed to identify these cases with detailed characteristics and present an extensive review of SIADH associated with escitalopram use which has not been included in the previous reviews. We reviewed literature published between January 1, 2002, and March 31, 2018, through search on Medline and found fourteen reported cases of SIADH associated with escitalopram use (Table 3). We also went through reference sections of each of these reports to identify other missing articles.

Nashoni et al. reported the first case of hyponatremia associated with escitalopram in 2004 in a 62-year-old female which occurred 3 weeks following initiation of escitalopram [8]. Majority of these reported escitalopram-induced SIADH were in females (64.28%). While age more than 65 years was found to be risk factor for SSRI-induced SIADH [2], this association was not seen with escitalopram, although the number of cases were limited to draw a definitive statistical conclusion. 50% of reported cases were in patients below 65 years of age. The youngest reported case was in a 47-year-old male who presented with seizures within 4 days of initiation of the drug while the oldest patient was a 97-year-old female [9, 10]. The mean serum sodium levels on presentation were 115 mEq/L.

The most common symptoms at presentation included confusion, seizures, and weakness. None of the reported cases had asymptomatic presentation which signifies underreporting of the association between escitalopram and SIADH. Similar to other SSRIs, the risk of hyponatremia is the highest during the first weeks with the earliest onset being within 2 days of starting escitalopram but can also occur even after 2 months of medication initiation [10, 11]. The median time of onset of symptoms was 7 days. Many of the patients reported in the literature were on multiple medications known to cause hyponatremia especially thiazide, proton pump inhibitor, and other psychiatric medications; however, a temporal association was drawn to escitalopram in all the cases [8, 9, 12–15]. Diken et al. reported a case of a 59-year-old male who underwent coronary artery bypass graft and developed hyponatremia with sodium levels of 107 mEq/L about 1 week following concomitant use of escitalopram 10 mg once daily and hydrochlorothiazide 50 mg once daily. Interestingly, this particular patient had prior history of use of escitalopram without any side effects [15].

The main modality of management in SIADH is water restriction, although in severe symptomatic cases hypertonic saline (3%) and drugs such as loop diuretics (furosemide), demeclocycline, and vaptans should be used [16]. Water restriction is the most important treatment modality in patients with SIADH. Those who do not respond fully or respond only partially can be treated with salt tablets and diuretics. Demeclocycline has been used previously which lead to acute kidney injury and hence its use has been limited. Vaptans, a selective V2 receptor antagonist (Tolvaptan and Conivaptan) can be safely used for treatment of SIADH in carefully monitored patients [17, 18]. High cost and overcorrection remain a potential risk for vaptans [18]. In all 14 cases, hyponatremia improved after discontinuation of the drug and active treatment for SIADH with fluid restriction and administration of hypertonic saline. However, in our case Tolvaptan was used for correction of hyponatremia. Rapid correction of hyponatremia leads to serious neurologic problems with the most severe including osmotic demyelination syndrome and hence close monitoring of sodium during the treatment of SIADH is of paramount importance [16, 19]. One patient had complication secondary to treatment of hyponatremia resulting in central pontine myelinolysis [20].

In 2012, Tsai et al. reported a case of 73-year-old female with history of dementia with Lewy bodies who developed delirium after being on escitalopram for 2 months. She was found to have serum sodium of 124 mEq/L on presentation. She recovered to baseline within 2 weeks following discontinuation of medication. Her delirium and hyponatremia (122 mEq/L), however, recurred after escitalopram was rechallenged. During the rechallenge phase, she had an early onset of symptomatic hyponatremia at 4 days [11]. This case signifies that rechallenge of escitalopram leads to SIADH with early initiation of the medication. The mean time for normalization of serum sodium levels after discontinuation of escitalopram and active treatment was found to be 5.8 days among the ten reported cases with data on resolution time. Mirtazapine was noted to be a good choice of antidepressant for those patients who developed escitalopram associated SIADH on review of literature [8, 14].

## 6. Conclusion

Our case highlights the clear association of escitalopram use with SIADH in the absence of any significant medical comorbidity or concomitant drug use. With our literature review of all the published case reports, we could infer that female gender and use of concomitant medication may be the risk factors for SIADH in patients taking escitalopram. However, more data is needed for obtaining meaningful conclusion regarding the risk factors and associations. Hyponatremia in most cases started within first week of treatment and resolved within 2 weeks after discontinuation of the medication. Patients being started on escitalopram and other SSRIs should be informed about this life-threatening adverse effect and the warning signs. Factors leading to overhydration like water intake for urinary tract infections and ingestion of excess water during exercise can lead to precipitation of SIADH [21]. We suggest regular serum electrolyte monitoring in patients receiving escitalopram and always evaluate patients for hyponatremia when presenting with symptoms of weakness, confusion, or seizures. Patient who once developed SIADH on escitalopram should not be ideally rechallenged with the same medication due to risk of causing more severe SIADH and may be started on alternative antidepressants like Mirtazapine.

## Figures and Tables

**Table 1:** Laboratory results at admission.

Laboratory Test	Levels	Reference Range
Serum Sodium	105 mEq/L	135 –145 mEq/L
Serum Chloride	74 mEq/L	99-109 mEq/L
Serum Osmolality	234mosm/kg	275–295 mosm/kg
Serum Creatinine	0.69 mg/dL	0.40-1.10 mg/dL
Blood Urea Nitrogen (BUN)	10 mg/dL	5-21 mg/dL
Urine Sodium	68 mEq/L	25-150 mEq/L
Urine Chloride	75 mEq/L	75-170 mEq/L
Urine Osmolality	468 mosm/kg	50-1400 mosm/kg
Morning Cortisol	49.2 mcg/dL	1.0-75.0 mcg/dL
Thyroid Stimulating Hormone (TSH)	0.86 mcIU/mL	0.5- 5.0 mcIU/mL

**Table 2:** Diagnostic criteria for the syndrome of inappropriate antidiuretic hormone secretion (SIADH).

**Essential criteria**

1. True plasma hypoosmolality (<275 mOsm/kg H2O)
2. Inappropriate urinary response to hypoosmolality (urine osmolality >100 mOsm/kg H2O)
3. Euvolemia; no edema, ascites, or signs of hypovolemia
4. Elevated urine sodium (>30 mEq/L) during normal sodium and water intake
5. No other causes of euvolemic hyponatremia

**Supplemental criteria**

1. No significant increase in serum sodium after volume expansion, but improvement with fluid restriction.
2. Unable to excrete >80% of a water load (20 cc/kg) in 4 hours and/or failure to achieve urine osmolality <1mOsm/kg H2O

**Table 3:** Summary of all reported cases of SIADH associated with escitalopram.

Case Report	Age	Sex	Escitalopram Dose	Onset (Days)	Sodium Levels (mEq/L)	Presenting Symptoms	Treatment given	Resolution (Weeks)	Comorbid conditions	Medications	Remarks
Nashoni et al, 2004 [8]	62	F	10 mg	21	110	Syncope	Medication discontinuation	1 week	Hypertension, Hyperlipidemia Atrial fibrillation Protein C deficiency Osteoporosis	Losartan Simvastatin Sotalol Warfarin Calcium Vitamin D	Patient was later treated with Mirtazapine for depression

Nirmalani et al, 2006 [22]	50	M	20 mg	28	121	Weakness Dizziness	Medication discontinuation Fluid Restriction	5 days	Depression with Psychotic features, Hypertension, COPD Osteoarthritis GERD	Risperidone	

Adiga et al, 2006 [12]	81	F	10 mg	21	120	Generalized weakness & Recurrent Falls	Medication discontinuation Hypertonic saline	1 week	Alzheimer's' disease Hypertension Osteoporosis	Ramipril Alendronic acid Donepezil Mirtazapine HCTZ	Patient noted to have Renal tubular defect

Grover et al, 2007 [14]	67	F	10 mg	28	127	Delirium	Medication discontinuation	4 weeks	Bipolar affective disorder Hypertension Diabetes mellitus	Sodium valproate HCTZ Gliclazide Aspirin Losartan	Patient was later treated with Mirtazapine & Valproate for Moderate depression

Grover et al, 2007 [14]	75	M	10 mg	10	126	Seizures	Medication discontinuation	2 weeks	Hypertension Generalized anxiety disorder	Atenolol Amlodipine	Patient was later treated with Mirtazapine for Generalized Anxiety disorder

Covyeou et al, 2007 [13]	75	F	Unknown	5	116	Unknown	Medication discontinuation	5 days	Hypertension	Amlodipine HCTZ Aspirin Omeprazole Alprazolam	

Koski et al, 2009 [9]	97	F	5 mg	7	113	Recurrent Falls & Confusion	Medication discontinuation Fluid restriction Hypertonic saline	Unknown	Hypertension, Anxiety UTI (diagnosed 1 day prior to hospitalization)	Tolterodine Atenolol Furosemide Lisinopril Docusate Ciprofloxacin (started 1 day prior to hospitalization)	

Tsai et al 2012 [11]	73	F	10 mg	>60	124 122	Delirium	Medication discontinuation Fluid restriction	2 weeks 1 week	Lewy body dementia	Trihexyphenidyl Bethanechol Tamsulosin	Failed rechallenge of escitalopram as patient developed Hyponatremia

Pae et al, 2013 [10]	47	M	5 mg	2	110	Seizure	Medication discontinuation	4 days	Quadriplegia with Spinal A-V Malformation Depression	No other medication	

Soysal et al, 2014 [23]	76	F	10mg	28	113	Confusion Lethargy Incontinence	Medication discontinuation Hypertonic saline	Unknown	Hypertension Diabetes mellitus, Alzheimer's disease Sleep disorder	Losartan Sitagliptin Rivastigmine Memantine Trazodone	

Diken et al, 2016 [15]	59	M	10 mg	7	107	Confusion, Hallucination, Drowsiness	Medication discontinuation Fluid restriction Hypertonic saline	4 days	COPD Diabetes mellitus	Aspirin, metoprolol perindopril amiodarone Spironolactone Hydrochlorothiazide	Recent introduction of hydrochlorothiazide

Parmar et al, 2016 [20]	50	M	10 mg	3	94	Seizure	Medication discontinuation	5 days	Hypertension Panic disorder	Telmisartan Aspirin	Developed central pontine myelinolysis from rapid correction of sodium

Rawal et al, 2017 [24]	54	F	Unknown	4	116	Seizure	Medication discontinuation Fluid restriction	Unknown	Hypertension Depression	Telmisartan Salt restriction	

Vidyasagar et al, 2017 [25]	58	F	Unknown	14	107	Severe Constipation	Medication discontinuation High salt diet Fluid restriction	Unknown	Seronegative spondyloarthropathy Diabetes mellitus Dysthymia	Prednisolone, Hydroxychloroquine Methotrexate Insulin	
